# Patient and public involvement in priority‐setting decisions in England's *Transforming* NHS: An interview study with Clinical Commissioning Groups in South London sustainability transformation partnerships

**DOI:** 10.1111/hex.12948

**Published:** 2019-08-14

**Authors:** Clare Coultas, Katharina Kieslich, Peter Littlejohns

**Affiliations:** ^1^ School of Population Health & Environmental Sciences, Faculty of Life Sciences & Medicine King's College London London UK; ^2^ Department of Political Science Universitat Wien Vienna Austria; ^3^ Present address: King's College London, Universitat Wien Vienna Austria

**Keywords:** activism, clinical commissioning groups, co‐production, England's National Health Service, integrated care systems, patient and public involvement, priority‐setting, Sustainability and Transformation Partnerships, trust

## Abstract

**Background:**

Patient and public involvement (PPI) in health‐care commissioning decisions has always been a contentious issue. However, the current moves towards Sustainability and Transformation Partnerships (STPs) in England's NHS are viewed as posing the risk of reducing the impact of current structures for PPI.

**Objective:**

To understand how different members in clinical commissioning groups (CCGs) understand PPI as currently functioning in their decision‐making practices, and the implications of the STPs for it.

**Design:**

Thematic analysis of 18 semi‐structured interviews with CCG governing body voting members (e.g. clinicians and lay members), non‐voting governing body members (e.g. Healthwatch representatives) and CCG staff with roles focussed on PPI, recruited from CCGs in South London STPs.

**Results:**

There are contestations amongst CCG members regarding not only what PPI is, but also the role that it currently plays and could play in commissioning decision making in the context of STPs. Three main themes were identified: PPI is ‘going out’ into the community; PPI as a disruptive power; and PPI as co‐production, a ‘utopian dream’?

**Conclusions:**

Long‐standing issues distinctive to PPI in NHS prioritization decisions are resurfacing with the moves towards STPs, particularly in relation to contradictions between the rhetoric of ‘partnership’ and reorganizations that foster more top‐down control. The interviews reveal pervasive distrusts across a number of levels that are counterproductive to the collaborations upon which STPs rely. And it is argued that such distrust and contestations will continue until a formalized space for PPI in STP priority‐setting is created.

## 1 BACKGROUND

England's National Health Service (NHS) is currently in a state of transformation centred on integrating the commissioning and provision of health and social care services, through place‐based partnerships. This process, started in 2016, has been set in motion without legislative backing, framed instead as locally driven changes to working relationships and cultures, aimed at fostering collaboration, trust and devolved budgetary power, with the aim of enhancing efficiency, responsiveness to local population needs and ending ‘fractured’ care.^1^, ^2^ Clinical commissioning groups (CCGs) (themselves only established in 2012) are GP‐led statutory bodies, whose role involves ‘assessing local needs, deciding priorities and strategies, and then buying services on behalf of the local population from providers such as hospitals, clinics, community health bodies etc…’.^3^ They have been a key site at which this drive towards integration has been initiated. Through a variety of arrangements, CCGs are now working collaboratively as Sustainability and Transformation Partnerships (STPs), in some cases with a view to evolving into integrated care systems (ICS), in which priorities and management of resources are agreed upon at a systems level, which, for instance in London, equates to the equivalent of 4‐6 CCGs in each of the five London STPs.^4^ From the outset however, STPs have been criticized for not sufficiently involving NHS staff, patients, publics and local government, and their non‐statutory nature has only heightened public distrust of the process and fears of creeping privatization.^5^, ^6^

Patient and public involvement (PPI) in the NHS has always been considered an important aspiration but remains a contentious issue, often lacking a clear rationale in regard to aims and means, along with evidence of outcomes.^7^ Nevertheless, whilst it has managed to maintain its position in successive NHS reorganizations, as Hudson argues, the current STP process in which commissioning decisions are functioning on larger scales (e.g. shared contracting, the formal merging of CCGs) poses the risk of sweeping current structures for PPI into irrelevance.^6^ In this interview study, carried out between October 2017 and May 2018, we look specifically at PPI in NHS priority‐setting decisions, seeking to understand how different CCG board members and staff covered by two South London STPs understand and observe PPI as functioning in practice, and the implications they perceive there being for PPI in STP commissioning. In so doing, we strive to elucidate practice‐based insights into the role[s] and associated challenges of PPI in commissioning decision making, and how different people and groups *doing* PPI are navigating the emerging STP process.

Activist campaigning for public representation in NHS spending and planning decisions dates back to the 1960s.^8^ The first formalized structures were the Community Health Councils (CHCs), which were created in 1974; however, PPI in priority‐setting decisions was only first legally articulated in the 2001 Health and Social Care Act (HSCA—Section 11). This policy section has survived subsequent policy amendments, and in its current form requires CCGs and NHS England (NHSE) to ‘make arrangements to secure that individuals to whom the services are being or may be provided are involved (whether by being *consulted or provided with information* or in other ways): a) in the planning… b) development and consideration of [change] proposals… and c) decisions’^9^ (p.50‐51 – italics added). NHSE use terms such as ‘involvement’, ‘engagement’, ‘consultation’ and ‘participation’ interchangeably^9^; however, Stewart identifies them as ‘synonyms of uncertain equivalence’ and tabulates the different scholarly typologies of PPI, all of which categorize terms according to the ‘level’ or degree of power gained by citizens.^10^ Notably, the terms used in policy—consulting and informing (italicized above)—are in what she describes as the ‘uncontroversial middle range’^10^ and signify citizen power that is a ‘level’ below the STP‐defining term ‘partnership’. Nevertheless, references to the *co‐design* of personalized health budgets (PHBs) for patients with chronic conditions in London STP plans do indicate partnership in commissioning, although the implementation of this being described as patient representatives sitting on CCG boards and committees^11^ does raise questions of whether the myriad of processual challenges to doing meaningful co‐design is appreciated.^12^ Earlier research is certainly indicative of there being wide disparities between the rhetoric and reality of PPI in commissioning self‐management.^13^

The King's Fund identifies STPs and ICS' as creating opportunities for advancements in PPI, for ‘joined‐up listening’ in which patient insights can be integrated across entire pathways of care.^14^ However, a historical lens indicates two main on‐going tensions that underlie PPI in priority‐setting in England's NHS and which need attention. The first relates to the ambiguous conceptualizations of PPI, owing to how state‐driven forms of public management that frame PPI in democratic terms as ‘voice’ have been conflated with market‐driven framings of PPI as ‘choice’.^15^, ^16^ In the public forums that replaced CHCs, this could be seen in how despite using the language of ‘partnership’ (indicating voice),^17^ ‘the public’ were used more as a ‘sounding board’, akin to market research, that legitimized pre‐defined policies and priorities, as opposed to having any real influence of their own.^18^ And today, the framing of patients with PHBs as ‘integrators’ of STPs^19^ also indicates this rhetoric of choice *as* empowerment, that Tritter identifies as dangerous in that health systems are needs‐ rather than wants‐based.^16^ Nevertheless, the legitimacy of democratic framings of citizen power in the NHS has from the outset also been called into question owing to issues of representativeness. 15, 20, 21, 22

The second tension relates to the shifting functions of PPI (both ‘invited’ and ‘uninvited’)^10^: PPI has been identified as holding the power of managers, health professionals and governments in check,^23^, ^24^, ^25^ although recent research in CCGs suggests that public knowledge is failing to permeate clinician power^26^; but also public activisms,^27^ ‘loss of confidence’^17^, ^21^ and disinterest^28^ have instigated changes to PPI structures. Nevertheless, Newman discusses how New Labour's ‘Third Way’ philosophy obscured the ‘publicness’ of public services and recast the public sphere as a ‘series of horizontal spaces’ in which citizenship and participation were localized, for instance on a specific service, rather than something that was held in common, displacing possibilities for wider justice and equality claims.^29^ And since then, research indicates that publics are struggling to mobilize, weakened by their not sharing a collective identity.^30^ Furthermore, since 2016, critiques of the top‐down ‘structuring’ of public engagements, along with managerial and clinician conceptualizations of PPI as a means of mediating and ‘softening oppositions’ in public opinion, focussed on legitimation, have returned.^6^, ^31^, ^32^, ^33^, ^34^

We therefore propose that a practice‐based understanding of what PPI in ‘new’ NHS decision making means is needed. For as Carter identifies, ‘Implementers’ interpretations of “new” policy, combined with their existing ritualised practices… weave imaginative visions of the future and sedimented historical beliefs into a unique local policy settlement’ (p.493).^35^ What are the current relational dynamics between managers, clinicians and publics, and how are these relationships adapting to the STP‐led changes to the ‘levels’ at which decision‐making happens; how is PPI understood and implemented by those overseeing and doing it in practice; and what are the implications of these for PPI in priority‐setting in England's transforming NHS?

## 2 METHODS

This paper draws on data that were collected as part of a larger qualitative study that sought to understand how a wide range of social values influence decision making in CCGs (of which [patient and public] participation was one).^36^ These values had been derived through a series of workshops which included local PPI representatives.^37^ Twelve CCGs, making up two STPs, in the South London area were approached through networks arising from the research team's place in the South London CLAHRC, and emails were sent out inviting members of the CCGs to participate. ‘Members’ in this study context are understood to include voting governing board members (e.g. clinicians, lay members), non‐voting governing board members (e.g. Healthwatch representatives) and CCG staff. As indicated in Table 1, eighteen interviews were carried out representing a range of CCG members (their specific CCG and STP affiliations are not specified for confidentiality purposes). The interviews were carried out by KK and CC between October 2017 and May 2018 and were digitally recorded and transcribed verbatim. Ethical approval for the study was obtained from the King's College London Research Ethics Committee, and all participants in the study gave informed written consent.

**Table 1:** Interviewee characteristics

CCG Position	Number of interviewees	PPI ‘Expert’ [10 + y]	CCG Decision‐making legitimate?
Clinician voting member	2 × GPs 1 × Nurse	–	3 × Yes
Lay voting member	3 × PPI focus 2 × Governance 1 × Finance	2	4 × Yes 2 × No owing to poor PPI
Healthwatch—Non‐Voting	6 × Managers 1 × Volunteer Overseer	2	2 × YES 5 × No owing to poor PPI
CCG Staff—Non‐Voting	2 × PPI Coordinators	1	1 × Yes 1 × No owing to poor PPI
TOTAL	18	5	11 × Yes / 7 × No

The interviews were semi‐structured and questions asked participants to first articulate how they understood a particular social value (e.g. accountability, transparency, participation, clinical effectiveness, cost‐effectiveness, fairness, quality of care), and then discuss their views on its influence in CCG decision making, giving examples when possible. No specific questions were asked about the STPs, yet they were discussed in all but three interviews. This paper only reports on the collected data pertaining to participation. The transcripts were analysed thematically using both a deductive and inductive approach,^38^ in which codes were inductively identified in relation to each of the social values deductively focussed on, but also for any values that were inductively identified. All interviews were double‐coded by KK and CC to ensure robust analysis, and themes were collaboratively identified through multiple discussions between KK, CC and PL. For the purposes of this paper, themes were searched for in all the coded sections of text that pertained to PPI, and developed by also maintaining a view of the different perspectives of the members’ positioned roles and experiences.

## 3 RESULTS

The interview analyses revealed contestations in understandings of not only what PPI is, but also the role that it currently plays and could play in commissioning decision making in the context of STPs. In some cases, these differences in understanding were related to the roles of interviewees, arguably indicative of the on‐going power tensions between managers, clinicians and publics. However, there were also interesting disagreements amongst the various non‐clinicians, and too, agreements that clearly counteracted both clinician and policy understandings of PPI. Three main themes were identified: PPI is ‘going out’ into the community; PPI as a disruptive power; and PPI in commissioning as co‐production, a ‘utopian dream’? Each will now be discussed in turn.

### 3.1 PPI is ‘Going Out’ into the community

There were fundamental disagreements between the clinicians and non‐clinicians (e.g.Lay Members [LM] , Healthwatch representatives and CCG staff) over what PPI is. The three clinicians described PPI in commissioning as LMs on the governing board, patient reference groups and consultations, viewed as serving the function of governance/oversight, and the testing of engagement plans and commissioning strategic priorities. Firstly, none of the non‐clinicians described LMs as PPI, and HW6 did not even view them as ‘the public’, describing the importance of the HW non‐voting seat on the board as at least keeping ‘*a bit of public representation in the room’*. Four of the LMs described themselves as having an oversight/governance role, in terms of ‘*protecting the doctors from themselves [in regard to conflicts of interest’* (LM3), and acting as a ‘*warrior battle lad [ensuring] that the decision‐making chain is always visible’* (LM4), whilst the remaining two LMs, both whose roles focussed on PPI, described their contribution more in terms of mediation. LM2 argued that their governance role was limited as the LMs did not have a voting majority, but stressed the importance of LMs in maintaining continuities with an ever‐changing CCG personnel, and diffusing misunderstandings between them and representatives of the LGA, voluntary sector and communities, ‘*it's a constant struggle’*. And LM1 focussed entirely on the importance of LMs for supporting conversations with the public in terms of ‘*communicating the totality of the [health] environment’* in relation to priority‐setting, in the hope of helping to diffuse partisanship.

The second difference with the clinicians' understandings of PPI was in the importance non‐clinicians placed on ‘going out’ into the community, in the light of the need to at least attempt at ‘representativeness’. Both of the CCG staff emphasized this and described their roles in terms of mediating between the CCG and community groups who were distrustful of CCGs, *‘The first thing we heard was, “somebody came and saw us three months ago, but we never saw them after that… what's going to be different in terms of your approach to keeping us involved?”’* (Staff1). Both described ‘chasing up’ commissioners to ‘touch base’ and feedback the outcomes of PPI, ‘*oftentimes it doesn't lead to a lot… [but] they can still be honest with the group’* (Staff2), and Staff1 described how ‘going out there’ was ‘*a learning experience for our commissioner managers who are not used to doing that in their normal roles’*. Many of the HW representatives however described commissioners as relying too much on them for PPI, often considering just having HW ‘at the table’ as sufficient, and the pressure that some felt because of this, ‘*a lot of our influence depends on how active we are able to be, and whether we're willing to ask awkward questions’* (HW5). And seven of the fifteen non‐clinicians questioned the legitimacy of current commissioning decisions outright, owing entirely to poor PPI practices, described as representing a ‘democratic deficit’ (HW3), as paternalistic (LM1) in that decision making is ‘*not a discursive process… [and] too much information acts as smokes and mirrors’* (HW6), and as merely a ‘sanity check’ as opposed to actually engaging with public and particularly opposing ‘voices’ (HW1, LM2, HM5, Staff1).

Where there was no difference between clinicians and non‐clinicians, was in the majority viewing that the STPs represented a ‘*shift back to the didactic central approach’* (Clin1), or how ‘*the central has just situated themselves a lot closer to the local so they're now overseeing people… [and controlling the agendas]’* (HW6). Not only was this viewed as a deprioritizing of PPI, but also a shutting down of dissent as LGAs and PPI were ‘brought in’ [to the centre]. This process was described as ‘*cumbersome people smoothing the heckles and getting everyone working together’* (LM5), and HM5 described how after having asked questions about whether STPs would be consulted on, and getting a ‘very shirty’ reply, ‘*I was like the sort of unpopular uncle that you stick on the table at the back. Because that is what they do. You have a name badge and you sit where your name badge is’*. The three interviewees who saw STPs as having a positive potential for PPI in decision making focussed on the networking capabilities of the local transformation boards, so that PPI ‘*communications and engagement [can be done] as one organisation, one approach’* (Staff1). Yet, HW3 did acknowledge that these networks could also make it difficult ‘*to tie the views that people gave you into decisions that are being made by different people, different structures [ie at an STP level]’*. LM1 certainly described how this ‘one organisation’ approach had felt very ‘us versus them’ in public consultations and had blocked dialogue, whilst LM2 highlighted the difficulties and efforts spent on forging such unity, ‘*patients and publics… can see and feel those tensions between the competing priorities [of NHSE and LGAs]… [and] It makes people feel cross, [because] where's the focus on what's actually being delivered?’*

### 3.2 PPI as a disruptive power

The disruptive powers of patients and publics were identified by many interviewees, albeit from quite different perspectives. The clinicians described how PPI was very effective in counteracting the managerial power of NHSE, ‘*they [NHSE] have had to shift their way of working a bit’* (Clin1), and how ‘*the lay member's presence… [has] helped to make sure that… the view from NHSE, as the regulator… [isn't given] more weight than … the views of others’* (Clin2). The Healthwatch interviewees instead represented the disruptive potentials of PPI at the level of CCGs, yet they differed in their approaches to this. Three of the interviewees positioned the power of Healthwatch, as representatives of the local public, firmly outside of and independent to the CCGs. The nature of their described power was therefore adversarial, ensuring that CCGs ‘come clean’ about their plans (HW7), by refusing to do contracted work with the CCG, ‘*it's essential that we are seen as independent’* (HW4), and how the NHSE’s rhetoric of ‘*“nothing about us without us”… can be thrown out every now and again… it's a useful peg… [that] reasserts where we need to be’* (HM5). The remaining four Healthwatch interviewees however viewed their role as establishing power inside CCGs that can then be ‘brought’ to community groups. In this framing, interviewees described having to ‘*constantly demonstrate our credibility’* (HW1), and market themselves to CCGs for PPI contracts in terms of their being able to provide *‘a degree of independence’* (HW3). A number of Healthwatch and LM interviewees did however identify that the type of influence that PPI has in CCGs is largely dependent on the leadership and their own personal commitment (or not) to PPI in decision making. An example of this pervasive yet silent power of clinician leaders could be seen across two of our interviews in which the same example of PPI in a priority‐setting decision was brought up—the proposed closure of a local facility:

*“we are delaying the decision because of what people have said [in the consultation] and we are now looking very specifically at the issues that people raised… so I see a sincere response to public concern being dealt with”*. [Lay Member]

*“it's contract is expiring and we don't want to continue with it. The decision has been made, we're going to shut it down… the locals are now saying, ‘Well, you know, it's really helpful for us up here.’ No, sorry, we can spend that million quid much better. It's pretty tokenistic because we know what they're going to say and we're going to ignore it anyhow. I'm sorry, that's naughty but… a lot of NHS consultations are like that”*. [Clinician]

Five of the seven Healthwatch interviewees discussed how NHSE's demands for cuts, framed in terms of value and efficiency, have made PPI ‘disingenuous’, and how the STPs really expose the limitations on CCG decision‐making autonomy, ‘*they make out that they're kind of doing something new and unique, but the reality is that every CCG is doing exactly the same thing… I*'*m slightly sympathetic because often they're told you won't get this pot of money unless you do this and this by then’* (HW2). And two of the LMs described how in recent consultations that they've been a part of, they have witnessed ‘*quite a lot of partisanship… [and] feeling that somehow commissioners and NHS managers are not to be trusted, and “we must stop anything being changed”’* (LM1). LM4 described this as ‘poisonous activism’: ‘*demonstrations in the meetings… placards, politicians turning up, giving speeches, questioning the personal motives of the people on the panel… I mean really nasty stuff. And when it gets down to that level, you feel that you're not actually having a rational discussion anymore’*.

### 3.3 PPI in Commissioning as Co‐Production, a ‘Utopian Dream’?

A number of the non‐clinicians identified how PPI in commissioning should mean ‘co‐production’, however in the same breath, questioned whether this could ever be a reality. Part of this doubt was related to the quick time frames that all interviewees referred to as limiting PPI and that many described as having gotten worse with the STPs: ‘*the pace at which things have to happen, you know, they're facing such financial difficulties… it's not easy to actually have meaningful engagement… [that would require things to] inevitably be slowed down… a kind of… utopian situation’* (HW4). But more than this, the particular difficulties of PPI in priority‐setting decisions were highlighted: ‘*there's no point in asking patients at a general level what sort of neurosurgical service they would like. You know, some of the stuff is really clinical and technical and you can get people involved in the quality of what they receive, but in terms of planning and commissioning it, well some is better, is easier than others. There's a lot of effort in maternity and cancer, where there's more widespread experience of it for instance’* (LM6). Healthwatch interviewees also identified how decisions and decision‐making processes are only getting more complex with the STPs, ‘*to most people the kind of decisions that have to be made at that level are pie of the sky’* (HW2), and ‘*Imagine me trying to explain to people… there's a decision made by the CCG board but that's not the end of it. There's a joint decision‐making board. And then the NHS might decide to change their mind about how much money they're going to give for this thing, so then it might change again.’… at what point do we say right, this has happened or is happening?… a lot of the time we don't even have time to understand things… We need the people doing it to say. They don't even always know either’* (HW6).

Two of the non‐clinicians raised a concern about CCG decision‐making processes in general, in that a lot of the influencing or ideas behind decisions ‘*happen in private in the discussions between the CCG managers and their GP representatives’* (HW7). As LM5 identified however, ‘*you can't, in a great big board meeting, discuss everything in minute detail. But if you feel that you've been excluded from the process whereby people were influenced, you could worry that it wasn't as democratic as it might appear around the table where people vote’*. Yet, they also went on to say that, being a new lay person ‘*who knew nothing about it beforehand, I’m personally reassured that it isn't just the case that a management team on a directive from NHSE can just steamroller something through. They can't. There's far too much power vested in, particularly in the GPs’* (LM5). And LM6 who had over 10 years of experience in NHS governance with a particular focus on PPI remarked that ‘*quite rightly many say no [PPI doesn't influence commissioning decisions]… [however] it's a lot better than it used to be… It gets heard’*. Two of the clinicians also identified that ‘*the public quite rightly criticises us… we only ask for their opinion at the very end’* (Clin2) but emphasized that they were as honest as they can be ‘with our public’ and that actually the democratic model such as seen in the LGAs was less likely to effect honesty: ‘*they will say stuff to me that they can't possibly say in public… A bit two‐faced… I mean if they say anything faintly to do with anything that could be interpreted as downgrading the local hospital they would never get elected again!’* (Clin1). Yet, the STPs do pose a potential problem for the capacity of CCGs to be honest with their publics, what LM5 calls the ‘darker side’ of STPs—that despite the emphasis on ‘partnerships’, ‘*inevitably there will be some decision‐making at a high level, which you need all CCGs to implement’*.

## 4 DISCUSSION AND CONCLUSION

These interviews are of course not representative of all CCGs. However, we do suggest that they draw attention to two interconnected issues in current practices of PPI in commissioning that not only complexify the tensions identified in the literature in regard to the constructions and functions of PPI. But also, if not addressed, it could pose a problem for efforts at ‘joined‐up listening’^14^ in STPs and ICS'. Firstly, the interviews highlight the complexity of PPI in priority‐setting, and that whilst institutionalized structuring appears to be waning in the moves towards STPs, non‐clinician commitments to PPI *as voice* in commissioning remain strong. And secondly, how this closing down of space for PPI in priority‐setting is likely contributing to the distrust which increasingly typifies relations within commissioning structures.

In relation to the first, we propose that the interviews reveal the particularities of PPI in commissioning, different from, for instance, service design and evaluation where those affected by changes to a service can be clearly identified, and likely to have experiential knowledge relevant to the decisions being made. In contrast, many of the decisions being made by CCGs are technical, ‘pie in the sky’, and with the STPs are becoming increasingly more complex in terms of chains of decision‐making processes. PPI in commissioning therefore requires strategic thought towards how the contexts to decisions are described and explained, and to whom; an enormously complex and political task, which the interviews would suggest, is currently often falling to individual CCG staff, lay member ingenuities and Healthwatch. Whilst the non‐clinician emphases on ‘going out’ into the community suggest a strong commitment to ensuring that different public ‘voices’ and democratic principles of on‐going dialogues and accountability are involved in priority‐setting decisions. The fact that the institutionalization of these processes relies heavily on individuals in CCG leadership and Healthwatch really highlights the precarity of PPI as ‘voice’ in commissioning. And the absence of such efforts at the STP level is particularly striking. For instance, in an NHSE document aimed at providing practical guidance for delivering the STPs, ‘participation, co‐production, and diversity’ is only discussed briefly and in general terms, with the examples given focussed solely on PPI in relation to services (as opposed to priority‐setting).^39^

The second aspect builds on current literature that highlights mistrust as an issue in large‐scale changes such as STPs,^40^ by revealing pervasive distrusts across a number of levels: of STPs by publics and CCGs; of CCGs by Healthwatch and community groups; of the GPs by lay members and Healthwatch; and even of Healthwatch by the public, visible in interviewees concerns about Healthwatch being seen as independent. Even when sympathy is expressed for people trying to do PPI in the current commissioning climate, an overarching distrust in the system clearly remains. This is crucial because trust is foundational to the collaborations and ‘collectivity’ that STPs rely on.^41^ At the local level, the CCG staff can be seen to be attempting the difficult work of ‘reticulists’,^41^ mediating conflicts and distrust between CCG managers and patients and publics with the aim of building networks across boundaries. And whilst the LTBs hold scope for horizontal networking between people doing PPI, such vertical networking, in which patients and publics are bridged with the STPs, thus far, remains absent. Therefore whilst ‘the centre’ has situated itself closer to the local for oversight of decision making, it remains at a distance from patients and publics, also symbolized in the example given of Healthwatch name tags being placed at the back of the room in an STP meeting. Research suggests that forms of ‘contestatory’ (or uninvited) participation increase when institutionalized structures for participation are lacking,^42^ and this is indicated in the interviews, in both the references to partisanship and ‘poisonous activism’, but also the distrusting relations within commissioning structures. For instance, the interviews also suggest that Healthwatch can become more adversarial, be it owing to a closing down of opportunity for voice at the level of STPs or by an unsupportive CCG leader.

These issues of distrust and the precarity of PPI as ‘voice’ in England's NHS are not in and of themselves new. For the constraints on PPI as ‘voice’ and triggers of distrust—top‐down directives, limits on time and resources, ‘behind closed doors’ influencing, and the complexities of decisions—have been there from the outset. They relate to what Klein has for decades described as the fundamental contradiction in the NHS: its centralized control borne out of considerations of equity, but which has more recently been co‐opted by regulatory agencies; and its devolution, encouraging the responsivity and accountability of local decision‐makers to their publics, yet which has also given rise to critiques of ‘postcode lotteries’.^43^, ^44^ Rather, we suggest that they are being felt more acutely now because the ‘rules of the game’ are changing: meaningful democratic, ‘invited’, participation relies on socioeconomic securities that enable at least a consensus on procedure.^45^ And in the current contexts of austerity measures, ‘post‐truth’ distrust of elites, and a health system that is clearly governed through a manager‐led market model whilst using the rhetoric of partnerships (once more),^46^ such securities are glaringly absent.

A comparative analysis of the UK’s four health systems highlights how the creation of trusting relationships and collaborative work is hardest in fragmented and unstable systems, such as England's NHS.^47^ Furthermore, a recent experiment aimed at creating a ‘space’ for co‐productive learning between researchers, clinicians, patients, carers, and managers also illustrated how even at a ‘distance’ from NHS spaces, the institutionalized scripts that structure power asymmetries are enormously difficult to disrupt.^48^ Nevertheless, both studies emphasize the essentiality of public support and the legitimization of ‘non‐professional’ knowledge for NHS transformations and collaborations, respectively, that in the current contestatory context requires specific attention.^47^, ^48^ It is therefore concerning that in the NHSE guidance for delivering the STPs, PPI is referenced in isolation from discussions on the need to apply ‘social movement principles’ in health and care, indicating that patients and publics are not perceived to be a part of this ‘collective agency’.^39^ Yet, the ‘collective’ and ‘movement’ aspects of the suggested transformation strategies are also themselves questionable. In that ‘framing the issues in ways that engage and mobilise the imagination… [and] continually refreshing the story’ (p.15‐16)^39^ reflects more of a top‐down public relations and marketing logic, that not only sustains power asymmetries, but is at odds with the solidarity, emancipatory goals and [self‐initiated] collectivizing enactments through which mobilization for pre‐figurative change takes place.^49^, ^50^, ^51^

Therefore in highlighting differences in how PPI in commissioning is understood in the context of STPs, this study reveals that whilst the creation of more formalized spaces for PPI at higher levels of STPs are needed, so too is practice‐based evidence on the relational dynamics that can initiate and sustain collaborations between different identity groups in England's NHS. Not only do STP leaders have to act as boundary spanners, bridging built‐in conflicts of interest between commissioners and providers, health and social care organizations, and different patients and publics who are becoming increasingly more contestatory, they must also somehow inspire trust in a system that at present lacks any form of socioeconomic security. This is a task that requires much more than ‘compelling narratives’ and inspirational leadership,^39^ and reasserts the residual power of patients and publics in England's NHS.

## CONFLICT OF INTEREST

N/A.

## Data Availability

N/A.
